# Search for QTL affecting the shape of the egg laying curve of the Japanese quail

**DOI:** 10.1186/1471-2156-7-26

**Published:** 2006-05-05

**Authors:** Francis Minvielle, Boniface B Kayang, Miho Inoue-Murayama, Mitsuru Miwa, Alain Vignal, David Gourichon, André Neau, Jean-Louis Monvoisin, Shin' ichi Ito

**Affiliations:** 1 Génétique et Diversité Animales, Institut National de la Recherche Agronomique, Centre de Jouy, 78352 Jouy-en-Josas, France; 2 Faculty of Applied Biological Sciences, Gifu University, Gifu 501–1193, Japan; 3 Génétique Cellulaire, Institut National de la Recherche Agronomique, Centre de Toulouse, 31326 Castanet-Tolosan, France; 4 Unité Expérimentale de Génétique Avicole, Institut National de la Recherche Agronomique, Centre de Tours, 37380 Nouzilly, France; 5 Département de Génétique Animale, Institut National de la Recherche Agronomique, Centre de Jouy, 78352 Jouy-en-Josas, France

## Abstract

**Background:**

Egg production is of critical importance in birds not only for their reproduction but also for human consumption as the egg is a highly nutritive and balanced food. Consequently, laying in poultry has been improved through selection to increase the total number of eggs laid per hen. This number is the cumulative result of the oviposition, a cyclic and repeated process which leads to a pattern over time (the egg laying curve) which can be modelled and described individually. Unlike the total egg number which compounds all variations, the shape of the curve gives information on the different phases of egg laying, and its genetic analysis using molecular markers might contribute to understand better the underlying mechanisms. The purpose of this study was to perform the first QTL search for traits involved in shaping the egg laying curve, in an F_2 _experiment with 359 female Japanese quail.

**Results:**

Eight QTL were found on five autosomes, and six of them could be directly associated with egg production traits, although none was significant at the genome-wide level. One of them (on CJA13) had an effect on the first part of the laying curve, before the production peak. Another one (on CJA06) was related to the central part of the curve when laying is maintained at a high level, and the four others (on CJA05, CJA10 and CJA14) acted on the last part of the curve where persistency is determinant. The QTL for the central part of the curve was mapped at the same position on CJA06 than a genome-wide significant QTL for total egg number detected previously in the same F_2_.

**Conclusion:**

Despite its limited scope (number of microsatellites, size of the phenotypic data set), this work has shown that it was possible to use the individual egg laying data collected daily to find new QTL which affect the shape of the egg laying curve. Beyond the present results, this new approach could also be applied to longitudinal traits in other species, like growth and lactation in ruminants, for which good marker coverage of the genome and theoretical models with a biological significance are available.

## Background

From the same natural oviposition cyclic process, wild birds generally lay only one or a few clutches of up to two dozen eggs per year [1] whereas quail [2] and chicken [3] from highly selected commercial or experimental lines may produce several hundred eggs over the same period. Yet, their egg production changes with time, increasing from the onset of egg laying to peak production, then plateauing and finally declining steadily. It may be described by drawing an egg laying curve which can be modelled by theoretical equations [e.g.: [4-7]] with biologically meaningful parameters. Another, more global, measure of egg production is the total number of laid eggs which has been extensively studied from the standpoint of quantitative genetics [8,9], and for which QTL were recently detected in chicken [10,11] and Japanese quail [12]. This trait, however, conveys little information for explaining the shape of the egg laying curve and its variation. On the contrary, estimations of the parameters of the individual egg laying curves fitted to the observed egg production might be used as phenotypes, and searched for underlying QTL, but this approach has not been tested until now.

The objective of the present work was to produce the first set of QTL detected in birds for the parameters which determine the shape of the egg laying curve. For that purpose, in an F_2 _experiment with two quail lines selected for early egg production [13] and for high duration of tonic immobility [14], which had led already to the detection of QTL for total egg number, age at first egg and clutch length [12], the individual egg laying curves were fitted to egg production using three theoretical models [4,6,7]. The Gifu microsatellite marker panel [15] and the microsatellite map [16] developed from the same F_2 _reference families were used to detect QTL for the parameters of the curves.

## Results

### Egg laying curves

**Figure 1:**
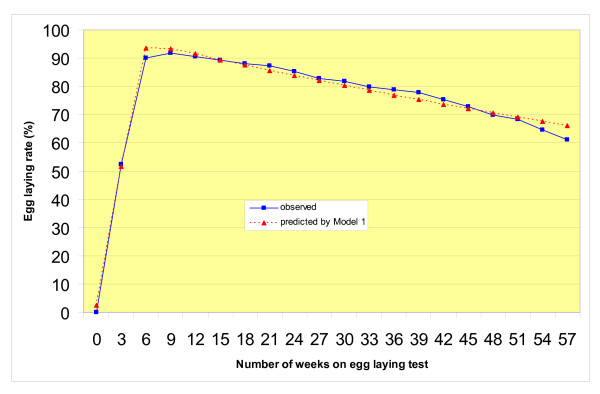
**Egg laying rate of F2 Japanese quail**. The blue solid line with squares represents the curve drawn from the mean F2 egg laying rates calculated from the individual egg production data. The red dashed line with triangles represents the theoretical egg laying rate curve adjusted to all individual F2 egg laying records using Model 1 [4].

**Figure 2:**
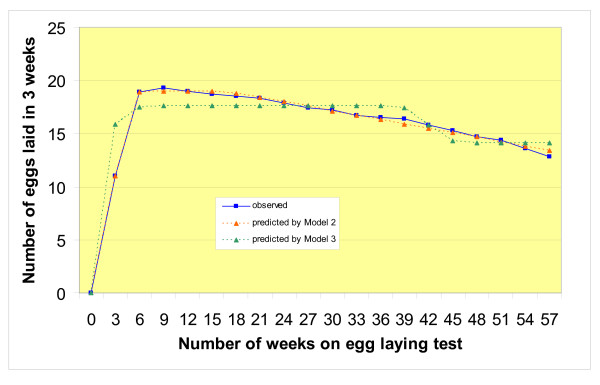
**Egg production of F2 Japanese quail**. The blue solid line with squares represents the curve drawn from the consecutive mean 3-week F2 egg productions calculated from the individual egg production data. The orange dashed line with triangles and the green dashed line with triangles represent the theoretical egg production curves adjusted to all individual F2 egg laying records using respectively Model 2 [6] and Model 3 [7].

Table 1 shows the equations of the three models, the estimations of their parameters, and their overall fit to the F_2 _data from the 359 female quail. It also lists the estimations of the same parameters for the two F_0 _lines (DD and LTI) used to produce the F_1 _generation, parent of the F_2_. Observed and predicted egg laying curves of the F_2 _population are in Figure 1 (Model 1 for egg laying rate) and Figure 2 (Models 2 and 3 for 3-week egg production).

**Table 1:** Egg laying equations of F2 and F0 (lines DD and LTI) Japanese quail populations using three theoretical models

Model 1 [4]	*elr*(*t*) = *a *× exp(-*bt*)/(1+exp(-*c*(*t*-*d*))				
Parameter	*a*	*b*	*c*	*d*	R^2^

F2	99.62 ± 0.77	0.00721 ± 0.00024	1.242 ± 0.129	2.901 ± 0.041	0.921
DD	97.55 ± 3.28	0.00899 ± 0.00138	1.022 ± 0.207	3.391 ± 0.177	0.880
LTI	93.24 ± 2.43	0.00734 ± 0.00106	1.253 ± 0.270	3.512 ± 0.154	0.927
Model 2 [6]	*y*(*t*) = (*y*_*P*/_*t*_2_) × *t *– *r *× (*y*_*P*/_*t*_2_) × Ln [(exp(*t/r*) + exp(*t*_2_/*r*))/(1 + exp(*t*_2_/*r*))] + *r *× *b*_4_× Ln [(exp(*t/r*) + exp((*t*_2_+*P*)/*r*))/(1 + exp((*t*_2_+*P*)/*r*))]				
Parameter	*y*_*P*_	*t*_2_	*b*_4_	*P*	R^2^
F2	18.98 ± 0.13	5.186 ± 0.124	-0.1385 ± 0.0055	11.58 ± 1.358	0.922
DD	18.18 ± 0.41	6.169 ± 0.370	-0.2014 ± 0.0401	11.53 ± 3.580	0.880
LTI	17.73 ± 0.28	6.307 ± 0.269	-0.1729 ± 0.0347	12.49 ± 3.132	0.926
Model 3 [7]	*y*(*t*) = [21*k*_1_× (1-exp(-*t*))/(1+exp(-*t*))] - [21(*k*_1_-*k*_2_)× (1 - exp(-*t*))/(1+exp(-(*t*-*c*_2_)))]				
Parameter	*k*_1_	*k*_2_	*c*_2_	-	R^2^
F2	0.8361 ± 0.0035	0.6712 ± 0.0057	42.01 ± 0.31	-	0.915
DD	0.8045 ± 0.0126	0.6925 ± 0.0215	29.25 ± 1.54	-	0.865
LTI	0.7802 ± 0.0095	0.6771 ± 0.0237	33.55 ± 1.35	-	0.906

^1^: *elr*(*t*) = egg laying rate and *y*(*t*) = number of eggs laid over the three previous weeks, after *t *weeks of egg laying test.

^2^: equations obtained with egg laying data from 359, 65 and 66 quail, in the F2 population and lines DD and LTI, respectively.

*a *= scale parameter*; b *= weekly rate of decrease in egg laying after peak; *c *= indicator of 1/variance in sexual maturity; *d *= number of weeks in test until sexual maturity.

*y*_*P *_= 3-week production at the egg laying plateau; *t*_*2 *_= number of weeks in test until egg laying plateau; *b*_4_= weekly rate of change in egg laying after the plateau; *P *= persistency of constant production.

*k*_1 _= proportion of peak production during the increasing phase of the curve; *k*_2_= proportion of peak production during the decreasing phase of the curve; *c*_2 _= duration of peak egg production.

Table 2 lists the parameters of the curves for which the detection of QTL was performed, and gives their biological meaning. It also shows the corresponding statistics (number, mean, and range) from the set of the individual curves adjusted to the data. Five, 7 and 51 curves could not be obtained by nonlinear regression under Models 1, 2 and 3, respectively.

**Table 2:** Statistics on the estimations of the parameters of the egg laying curves adjusted individually to the F2 Japanese quail and used as quantitative traits in the genome scan

Curve type	Trait (definition)	Mean	Standard deviation	Minimum	Maximum
Model 1 [4] (N = 354)	*b *(weekly rate of decrease in egg laying after peak)	0.0160	0.0416	-0.0068	0.3654
	*c *(indicator of 1/variance in sexual maturity)	3.223	3.155	0.1665	22.20
	*d *(number of weeks in test until sexual maturity)	4.186	4.842	0.6212	46.33
Model 2 [6] (N = 352)	*y*_*P*_(3-week production at the egg laying plateau)	18.98	1.615	8.329	23.46
	*t*_*2 *_(number of weeks in test until egg laying plateau)	5.272	1.935	2.299	20.25
	*b*_4_(weekly rate of change in egg laying after the plateau)	-0.3497	0.5767	-4.698	0
Model 3 [7] (N = 308)	*k*_1 _(proportion of peak production during the increasing phase of the curve)	0.8530	0.0944	0.3500	1.000
	*k*_2_(proportion of peak production during the decreasing phase of the curve)	0.6056	0.3082	0	0.992
	*c*_2 _(duration of peak egg production)	37.38	10.08	4.779	56.61

### QTL analysis

Table 3 shows the location of the chromosome-wide significant QTL, their position on the chromosome, the maximum F value obtained at this position, their genetic effects, the reduction of the residual variance obtained by fitting a QTL at this location, and the corresponding genome-wide significance.

**Table 3:** Chromosomal location, test statistic (F), genetic effects and significance of the QTL detected in the Japanese quail for the parameters of the egg laying curve

Curve type	Chromosome (map length cM)	Trait^1^	Position (cM)	Flanking markers	F	Additive effect ± SE	Dominance effect ± SE	Reduction of σ^2 ^(%)	Chromosome-wide probability	Genome-wide significance
Model 1 [4]	CJA10 (29)	*b*	29	*GUJ0085*	3.41	ns	-0.0712 ± 0.0275	1.4	0.05	ns
		*c*	29	*GUJ0085*	4.96	-0.936 ± 0.365	4.04 ± 2.07	2.2	0.01	suggestive
		*d*	29	*GUJ0085*	3.61	ns	-8.56 ± 3.20	1.5	0.05	ns
	CJA14 (8)	*b*	0	*GUJ0023*	3.23	ns	-0.0099 ± 0.0049	1.3	0.05	ns
Model 2 [6]	CJA06 (74)	*y*_*P*_	32	*GUJ0087*	4.05	0.362 ± 0.147	ns	1.7	0.06	ns
	CJA13 (36)	*t*_2_	2	*GUJ0005–GUJ0033*	4.61	0.542 ± 0.197	ns	2.0	0.04	ns
Model 3 [7]	CJA05 (21)	*k*_2_	9	*GUJ0059- GUJ0049*	3.71	ns	-0.1035 ± 0.0424	1.7	0.04	ns
	CJA10 (29)	*k*_2_	1	*GUJ0010–GUJ0085*	3.83	ns	0.1989 ± 0.0861	1.8	0.04	ns

^1^: Traits are defined in Table 2 and in the text.

ns = not significant.

Out of the 8 QTL found to be chromosome-wide significant (p_*c*_<0.05) on 5 of the 12 autosomes scanned in this study, one was genome-wide suggestive (p_*g *_<0.20) and none was genome-wide significant (p_*g *_<0.05). One chromosome-wide significant QTL for the parameter *t*_*2 *_related to the ascending phase of the egg laying curve in Model 2 was found on CJA13. Another QTL was close to chromosome-wide significance on CJA06 for the parameter *y*_*P *_which describes the plateaued phase of the curve in the same model. Chromosome-wide significant QTL for the parameters associated with persistency in Models 1 and 3 were found on CJA10 and CJA14 for *b*, and on CJA05 and CJA10 for *k*_2_. QTL were also found on CJA10 for the two parameters, *c *and *d*, of Model 1 which are related to sexual maturity.

In most cases, the genetic effect of the QTL was dominant, but the QTL was strictly additive in two instances, for *y*_*P *_and *t*_*2*_. The reduction of the residual variance due to fitting a QTL was small and did not exceed 2.2%.

## Discussion

### Egg laying curves

For all three populations, the F_2 _and the two F_0 _lines, the three models provided a good overall fit (R^2^) to the egg laying data as they accounted for around 90% of the total variation. Similar figures have been obtained on quail with Model 1 [17], but it seems that Model 3 underestimated the peak production of the F_2 _(Figure 2). Moreover, it was more difficult to adjust this model to individual curves as indicated by the high proportion (14%) of instances for which the numerical estimation of the parameters did not converge to an acceptable set of values. Of course, the coefficient of determination R^2 ^of individual curves fitted for each F_2 _quail was variable and often lower (in the range 99–58%) than for the global curve. Correspondingly, the variation of the biological parameters of the individual curves was much larger because they were estimated from 19 "observations" or less (only one value by bird and 3-week period). Because of the individual between-bird variations in the actual shape of the laying curves, it might be difficult to achieve more uniform goodness of fit for the within-bird curve parameters than that obtained in the present study, and as observed also with chicken [6,7]. This means that the precision on the phenotypes (the estimated parameters of the curve) used for the QTL analysis varied between birds, possibly making the detection of QTL more difficult to achieve than for traits measured directly.

### QTL

The majority of the chromosome-wide significant QTL corresponding to different parts of the laying curve obtained for the three models showed some dominance, which could be expected since there is consistent heterosis for egg production in poultry [18]. The significant genome-wide additive QTL for total egg number found in the previous analysis of the same set of quail [12] was mapped on CJA06 at the same position as the additive QTL found for *y*_*p *_(the number of eggs laid in 3 weeks at the time of maximum egg laying) in the present study. It is unlikely this convergence was coincidental, and it is an indication that the QTL which were only detected at the chromosome-wide level in the present work might truly correspond to regions with genes with some effect on the shape of the laying curve. Also, it is interesting to notice that the QTL analysis of the curves obtained under Model 1(for egg laying rate) and Model 3 (for 3-week egg number) led to the detection of a QTL for the decreasing phase of the curve (related to persistency) on CJA10. To our knowledge, there is no published report on similar QTL analyses of curves fitted to repeated observations of the same phenotype. Yet, this approach might be a good alternative to splitting the egg production into consecutive periods and searching for QTL for each period [11].

## Conclusion

For the first time, a genome scan was carried out to detect QTL for the parameters of curves modelling a biological process estimated by nonlinear regression and used as phenotypes for the analysis. The potential interest of this approach is in the integration of all the information to discover genes which affect the expression of the trait through time, as illustrated in the present work by the egg laying curve. Only chromosome-wide QTL were obtained in this study, which might be related to the limited value of the available models for describing accurately the individual egg laying curve and to the "imprecise" nature of the statistics used as a phenotype, and could possibly be overcome by using improved models and a larger data set. In any case, the approach is interesting, as a QTL for the maximum level of production reached for one of the curves was located at the same position as a genome-wide significant QTL previously reported for the total egg number. Of course, the results should be further tested on chicken and quail for egg laying. Interestingly, similar work might be carried out on longitudinal data in other species, like the growth curve or the lactation curve in ruminants.

## Methods

### Quail and husbandry

Two lines, LTI and DD, with different origins [19,20] and selected for early egg production [13] or for high duration of tonic immobility [14] were crossed reciprocally (12 single-pair matings) to produce the F_1 _generation. Ten males and 30 egg-laying females were selected at random across the F_1 _families to produce the F_2 _generation. Each F_1 _sire was mated to three full sisters from another F_1 _family to obtain 434 F_2 _female chicks (39 to 45 birds per sire family and 12 to 19 quail per full-sib family), in three consecutive hatches. The 57–week egg laying test analyzed in this work was completed by 359 birds.

The F_2 _quail were given successively standard starter (2901 kcal ME/kg, 11.5% moisture, 7% ash, 27% total protein, 8% fat and 4% crude fiber) and commercial layer (2709 kcal ME/kg, 11.5% moisture, 12% ash, 20% total protein, 4% fat and 4% crude fiber) diets. At 5 weeks of age they were assigned at random to individual cages of a 4-tier battery maintained at 22°C, in which they remained until the end of the experiment with free access to feed and drinking water.

### Traits

Egg production was recorded daily and individually from 5 weeks of age onwards. Global egg laying curves for all the F_2 _(and F_0_) quail and individual curves fitted to the 19 consecutive 3-week egg production of each F_2 _quail were obtained under three different nonlinear theoretical models [4,6,7] of egg laying. The parameters of the models with a biological meaning related to egg production were chosen as traits for which QTL analyses were carried out. For each model, the phenotypes were the estimates of the parameters of the individual laying curves.

Model 1 was: *elr(t) = a exp(-bt)/(1+exp(-c(t-d)))*, where *t *is the number of weeks in test, *elr *is the egg laying rate (%), *a *is a scale parameter, *b *is the rate of decrease of egg laying, 1/*c *is an indicator of the variance in sexual maturity, and *d *is the mean number of weeks in test until sexual maturity [4]. The traits for which QTL were sought under this model were *b*, *c*, and *d*. QTL analysis was not carried out on *a *because this parameter was highly correlated to *b *(r = 0.89) and has no biological interpretation.

Model 2 was: *y*(*t*) = (*y*_*P*/_*t*_2_)*t *- *r *(*y*_*P*/_*t*_2_) Ln [(exp(*t*/*r*) + exp(*t*_2_/*r*))/(1+exp(*t*_2_/*r*))] + *r b*_4 _Ln [(exp(*t*/*r*) + exp((*t*_2_+*P*)/*r*))/(1+ exp((*t*_2_+*P*)/*r*))], where *t *is the number of weeks in test, *y *is the number of eggs laid over the last 3 weeks, *r *is the duration of transition and was taken equal to 0.3 as in [6], *y*_*P *_is the level of constant production (that is, the 3-week production at the egg laying plateau), *t*_*2 *_is the time at transition from rapid increase to constant egg production (that is, the number of weeks in test until the egg laying plateau), *b*_4 _is the rate of decline in production (that is, the weekly rate of change in egg laying after the plateau) and *P *is the persistency of constant production (that is, the duration of the plateau, in weeks) [6]. The traits for which QTL were sought under this model were *y*_*P*_, *t*_2_, and *b*_4_. QTL analysis was not carried out on *P *because it was highly correlated to *y*_*P *_(r = 0.71).

Model 3 was: *y*(*t*) = [21*k*_1 _(1 - exp(-*t*))/(1+exp(-*t*))] - [21(*k*_1_*-k*_2_) (1 - exp(-*t*))/(1+exp(-(*t*-*c*_2_)))], where *t *is the number of weeks in test, *y *is the number of eggs laid over the last 3 weeks, *k*_1 _is the proportion of maximum production for the increasing phase of the curve, *k*_2 _is the proportion of maximum production for the decreasing phase of the curve, and *c*_2 _is the point of inflexion from the upper level of the increasing phase to the lower level of the decreasing phase of the curve (that is, a measure of persistency of egg production) [7]. The traits for which QTL were sought under this model were *k*_1_, *k*_2_, and *c*_2_.

The estimations of the parameters were obtained using the NLIN procedure of the SAS software [21]. Bounds were imposed on estimates of *b*_4 _(≤ 0), *k*_1 _(≤ 1) and *k*_2 _(≥ 0) to insure the coherence between these estimates and the biological meaning of the corresponding parameters of the curve. Under Models 1 and 2, individual egg laying curves could not be estimated for 5 and 7 quail, respectively, because convergence was not reached after 400 iterations, despite trying multiple starting values and using the Marquardt method. This problem was more severe under Model 3, however, for which 51 individual curves could not be obtained.

### Genotyping

Genotyping was carried out at Gifu University, and all quail were typed for the 72 microsatellites listed in the Gifu augmented panel [15]. The genotypic data were validated and stored in the MAPGENA database. All 24 F_0_, 40 F_1 _and 434 F_2 _quail in the present experiment belonged to the resource family set up to establish the first microsatellite quail map [16] built from 72 loci resolved into 13 linkage groups, and to carry out a previous QTL study of the same population [12]. Since then, additional work [22-24] made it possible to assign all 13 linkage groups (total map distance of 576 cM with a 10 cM average spacing between loci) to chromosomes. Consequently, the unassigned linkage groups Q03, Q04, Q08, Q09, Q10 and Q11 of the first map have been renamed respectively, CJA03, CJA13, CJA09, CJA04, CJA20 and CJA10 in the present work. The sex chromosome was not included in the present study.

### QTL analyses

Standard QTL detection was performed using the line cross method [25] implemented in the QTL Express software [26], and it took into account a hatch effect (3 classes). Five thousand permutations [27] were carried out to set significance levels (p_*c*_) for the most likely chromosome-wide QTL. Genome-wide significance (p_*g*_) for a QTL detected on a given chromosome was obtained from p_*c *_by: p_*g *_= 1-(1-p_*c*_)^1/*r*^, where r was the ratio of the length of this chromosome over the total chromosome length spanned by the present study. Chromosome-wide significance was set up at p_*c *_= 0.05. Genome-wide significant and suggestive thresholds were set up respectively at p_*g *_= 0.05 and 0.20.

## Authors' contributions

FM coordinated the study, analyzed the laying curves, and wrote the paper. BBK, SI, MIM and MM designed, organized, and conducted all the microsatellite work. AV led the mapping part. AN was responsible for the data bank, and DG and JLM supervised and carried out the data collection.
